# Visual Acuity and Retinal Thickness and Sensitivity after Intravitreal Ranibizumab Injection for Macular Edema in Branch Retinal Vein Occlusion

**DOI:** 10.3390/jcm13092490

**Published:** 2024-04-24

**Authors:** Ryota Nonaka, Hidetaka Noma, Kanako Yasuda, Shotaro Sasaki, Hiroshi Goto, Masahiko Shimura

**Affiliations:** 1 Department of Ophthalmology, Hachioji Medical Center, Tokyo Medical University, Tokyo 193-0998, Japan; ehiidvs@tokyo-med.ac.jp (R.N.); kana6723@tokyo-med.ac.jp (K.Y.); sasaki.shotaro.4m@tokyo-med.ac.jp (S.S.); mshimura@tokyo-med.ac.jp (M.S.); 2 Department of Ophthalmology, Tokyo Medical University, Tokyo 160-8402, Japan; goto1115@tokyo-med.ac.jp

**Keywords:** branch retinal vein occlusion, macular edema, ranibizumab, retinal sensitivity

## Abstract

**Background/Objectives**: To investigate changes in visual acuity and retinal sensitivity and thickness after intravitreal ranibizumab injection (IRI) for macular edema in branch retinal vein occlusion (BRVO) patients. **Methods**: This study evaluated 34 patients with treatment-naïve BRVO and at least 6 months’ follow-up after pro re nata IRI. Best-corrected visual acuity (BCVA) was determined as the logarithm of the minimum angle of resolution (logMAR). In nine retinal regions, retinal sensitivity was calculated by MP-3 microperimetry; and in nine macular subfields, retinal thickness was measured by optical coherence tomography (OCT); evaluations were performed before IRI and then monthly for 6 months. **Results**: IRI significantly improved visual acuity and retinal sensitivity and thickness. In patients with good improvement in BCVA (change in logMAR > 0.2), IRI significantly improved retinal sensitivity in eight of nine regions, i.e., in all except the outer non-occluded region, and in patients with poor improvement in BCVA (change in logMAR < 0.2), in six of nine regions, i.e., not in the inner, outer non-occluded, and outer temporal regions. We found significant differences in the trend profile in the foveal, outer occluded, and inner nasal regions between patients with good and poor improvement in BCVA. **Conclusions**: The findings suggest that IRI improves visual acuity and retinal sensitivity and thickness and that retinal effects may vary between patients with good and poor visual improvement.

## 1. Introduction

Branch retinal vein occlusion (BRVO) is a very common retinal vascular condition that occurs in patients with lifestyle-related diseases, including hypertension and arteriosclerosis. It affects people over the age of 40 years and has an incidence rate of 2%. Currently, 14 million patients have been diagnosed with BRVO worldwide [1,2]. Macular edema is the main cause of visual impairment in BRVO. Various treatment approaches were evaluated, including intravitreal triamcinolone acetonide injection and laser photocoagulation, but the outcomes were not satisfactory [3]. However, the management of macular edema was revolutionized by the development of anti-VEGF treatment [4,5]. After injection of anti-VEGF agents, macular edema improves dramatically, suggesting that VEGF plays a central role in this condition. 

In BRVO, macular edema poses the greatest threat to vision [6]. BRVO-related macular edema results from damage to the barrier between the retina and blood vessel endothelia due to increased intravascular pressure and decreased macular capillary blood flow [7]. The pathogenesis of macular edema involves vascular endothelial growth factor (VEGF) [7,8]. Besides visual acuity, studies in BRVO patients have investigated metamorphopsia [9,10], reading speed [11], contrast sensitivity [12], and vision-related quality of life (QOL) [13].

After the resolution of macular edema, visual acuity recovers to a relatively good level in most but not all eyes. Visual function is better represented by retinal sensitivity than visual acuity because retinal sensitivity (measured by MP-3 microperimetry) reflects function in both the fovea and the larger macular area, whereas visual acuity (measured by optical coherence tomography [OCT]) reflects only foveal function [14]. The following findings also suggest that visual acuity does not fully describe visual function after treatment of macular edema in BRVO patients, suggesting the importance of measuring retinal sensitivity: (1) Patients with good visual acuity sometimes have difficulty seeing certain objects, and patients with poor visual acuity sometimes have no difficulty [15]; (2) some patients can see things even though they have poor central fixation [16]; and (3) some BRVO patients show no improvement in visual acuity after macular edema improves [17].

Retinal sensitivity is affected by retinal disorders, including age-related macular degeneration [18,19], rhegmatogenous retinal detachment [20,21], diabetic macular edema [22], macular hole [14,23,24], and epiretinal membrane [25,26]. A few studies have reported on treatment-related improvements in retinal sensitivity in BRVO: Retinal sensitivity was improved by intravitreal triamcinolone acetonide injection in patients with BRVO and other types of retinal vein occlusion [27] and by intravitreal bevacizumab injection [28,29], intravitreal ranibizumab injection (IRI) [30,31,32,33], and intravitreal aflibercept injection [30,32,34] in patients with BRVO. However, none of these studies assessed retinal sensitivity at monthly intervals. Furthermore, it remains unknown whether there are differences in the improvement in retinal sensitivity between patients with good and poor visual improvement after anti-VEGF drug treatment. 

Previously, we reported on relationships among visual acuity, retinal sensitivity, and retinal thickness in patients with BRVO and macular edema for each of the nine macular subfields on retinal maps obtained by OCT [35]. As mentioned above, visual acuity reflects mainly foveal function; however, BRVO also affects the macular region and peripheral retina. The increase in vessel permeability in BRVO increases retinal thickness, particularly in the macular region and peripheral retina, because of their numerous capillaries. Accordingly, in the present study, we aimed to retrospectively investigate changes in visual acuity and retinal sensitivity and thickness after IRI for macular edema in BRVO patients in the nine retinal subfields. Furthermore, we aimed to compare changes in retinal sensitivity and thickness after IRI in patients with good and poor improvement in visual acuity.

## 2. Materials and Methods

### 2.1. Participants

We evaluated consecutive treatment-naïve patients with severe BRVO and macular edema-related visual impairment at the Department of Ophthalmology, Hachioji Medical Center, Tokyo Medical University, Tokyo, Japan. Patients were included if they had no history of any type of ocular treatment; were older than 30 years; had been examined within 3 months of noticing the first symptoms; had a central macular thickness (CMT) of at least 300 μm on OCT; had a maximum logarithm of the minimum angle of resolution best corrected visual acuity (logMAR BCVA) of 25 out of 30 as measured with the decimal visual acuity (Landolt chart) and the logMAR chart (5 m; NEITZ LVC-10, Tokyo, Japan); and had a post-IRI follow-up period of at least 6 months. Patients were excluded if they had any of the following: a concurrent ocular disorder (apart from mild disorders of refraction); a history of fundus laser therapy or triamcinolone acetonide injection, dexamethasone vitreous implants, or intravitreal injections of anti-VEGF drugs; other eye diseases that can cause macular edema or increase the concentration of VEGF in the eye (such as uveitis, neovascular glaucoma, wet age-related macular degeneration, and diabetic macular edema); eye diseases that can seriously affect vision (such as various types of glaucoma, corneal disease, and retinal detachment); contraindications to systemic or local surgery; or a history of internal eye surgery (such as cataract surgery). They were also excluded if they underwent internal eye surgery during the 6-month follow-up.

Before IRI, diagnoses were confirmed by a detailed ocular evaluation, including fundus and spectral-domain OCT (SD-OCT). Then, IRI (Lucentis; 0.5 mg in 0.05 ml; Genentech, Inc., South San Francisco, CA, USA) was performed by insertion of a 30 G needle through the pars plana, 3.5 mm posterior to the limbus. For the next 6 months, monthly comprehensive ocular examinations were performed that included measurement of BCVA. At these visits, IRI was administered pro re nata, i.e., IRI was repeated if OCT measured a CMT of at least 300 μm, including serous retinal detachment (SRD). Recurrence was assessed according to the number of times IRI was repeated.

### 2.2. OCT

**Figure 1:**
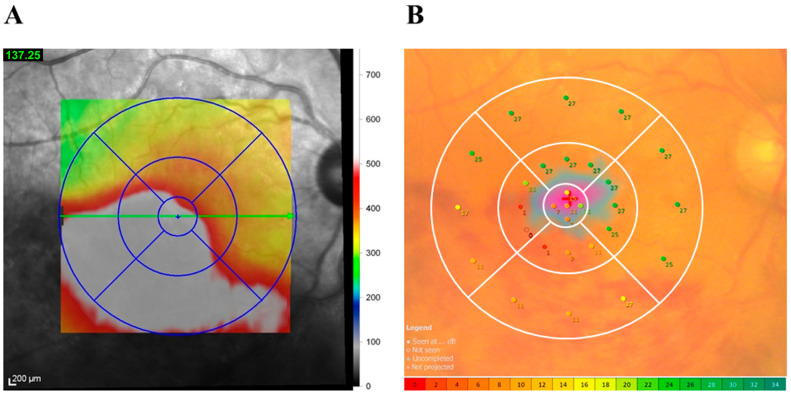
Measurement of retinal thickness and sensitivity in patients with branch retinal vein occlusion with macular edema. (**A**) Optical coherence tomography (OCT) map of retinal thickness in an example patient. Each map comprised 9 macular subfields: (1) fovea, (2) inner occluded, (3) outer occluded, (4) inner non-occluded, (5) outer non-occluded, (6) inner nasal, (7) outer nasal, (8) inner temporal, and (9) outer temporal. The dimeters of the circles were follows: central, 1 mm (4° × 4°); inner, 3 mm (10° × 10°); and outer, 6 mm (20° × 20°). (**B**) MP-3 microperimetry map of retinal sensitivity in an example patient. The figure shows the retinal sensitivity (mean) in the 9 macular subfields described in (**A**). The MP-3 program evaluated 29 points in the inner and outer retinal areas of the OCT map and 5 points in the fovea. The retinal area was divided into 9 regions: (1) fovea, (2) inner occluded, (3) outer occluded, (4) inner non-occluded, (5) outer non-occluded, (6) inner nasal, (7) outer nasal, (8) inner temporal, and (9) outer temporal. Mean retinal sensitivity was calculated for each region.

OCT (Spectralis OCT, Heidelberg Engineering, Heidelberg, Germany) and the associated software were used to produce retinal mapping images. Images were used to determine whether SRD was present [36]. The Spectralis software automatically calculated CMT, i.e., the thickness of the 1 mm field at the center of the OCT mapping image and the distance of the retinal pigment epithelium (including SRD, if present) from the inner limiting membrane. We assessed retinal thickness in the following 9 macular subfields (total area, 6 × 6 mm [20° × 20°]): the fovea subfield; occluded and non-occluded inner and outer subfields; nasal inner and outer subfields; and non-occluded inner and temporal inner and outer subfields [35] (Figure 1A). The Spectralis software also calculated the mean retinal thickness of the macular subfields. As a final step, the upper and lower inferior vein occlusion values were switched so that they matched the occlusion region.

### 2.3. Functional Mapping by Microperimetry

Microperimetry, a combination of digital fundus imaging and automated perimetry, was performed at baseline and monthly for 6 months thereafter with an MP-3 microperimeter (Nidek, Gamagori, Japan). A requirement of the MP-3 microperimeter is that pupil diameters exceed 4 mm, which was confirmed to be the case in all patients. MP-3 measurements used a 4-2 full threshold staircase strategy and standard Goldmann III stimulus. The MP-3 device has a maximum luminance of 10,000 asb and a stimulus dynamic range of 0 to 34 dB; the background luminance in the current study was 31.4 asb. The fixation target size was adapted to visual acuity in each patient, and the MP-3 device compensated for refractive errors [37].

The MP-3 microperimeter was used to create a retinal sensitivity map of the macula (central 20 degrees) in each eye. For the procedure, retinal sensitivity was determined by applying a stimulus at 29 locations in the retina (Figure 1B) [38,39]. The mean sensitivity was calculated before IRI and at each of the 6 follow-up months for 9 retinal regions: (1) foveal region, (2) inner occluded region, (3) outer occluded region, (4) inner non-occluded region, (5) outer non-occluded region, (6) inner nasal region, (7) outer nasal region, (8) inner temporal region, and (9) outer temporal region (Figure 1B). As with OCT, as a final step, the upper and lower vein occlusion values were switched so that they matched the occlusion region.

### 2.4. Statistical Analysis

Data were analyzed with SAS System 9.4 (SAS Institute Inc., Cary, NC, USA). Results are shown as mean ± SD or frequencies. Unpaired continuous variables were analyzed by an unpaired Student’s *t* test, and paired continuous variables were analyzed by a paired Student’s *t* test. One-way repeated-measures analysis of variance (ANOVA) was used to assess changes in retinal sensitivity and retinal thickness over time in 9 macular subfields and 9 retinal regions, respectively. Additionally, two-way repeated measures ANOVA was used to assess differences in changes in retinal sensitivity and retinal thickness over time between the group with improved visual acuity and those with poor visual acuity. A statistically significant difference was defined as a two-tailed *p* value below 0.05.

## 3. Results

### 3.1. Baseline Characteristics

Data were obtained from 34 eyes in 34 male and female patients with macular edema due to BRVO. Nineteen participants had occlusion of the superior vein, and 15 had occlusion of the inferior vein. Participant demographics and clinical characteristics are presented in Table 1. The majority of patients had systemic hypertension, and almost half had hyperlipidemia. SRD was present in half the patients before IRI and in 8 (23.5%) at the 6-month follow-up. The mean number of IRIs in the 6-month follow-up period was 2.7 ± 0.9. BCVA improved significantly over the follow-up period (1 month, 0.13 ± 0.18; 2 months, 0.17 ± 0.27; 3 months, 0.17 ± 0.26; 4 months, 0.15 ± 0.23; 5 months, 0.12 ± 0.23; and 6 months, 0.14 ± 0.25).

### 3.2. Effects of IRI on Retinal Thickness and Sensitivity in Patients with Branch Retinal Vein Occlusion and Macular Edema

**Figure 2:**
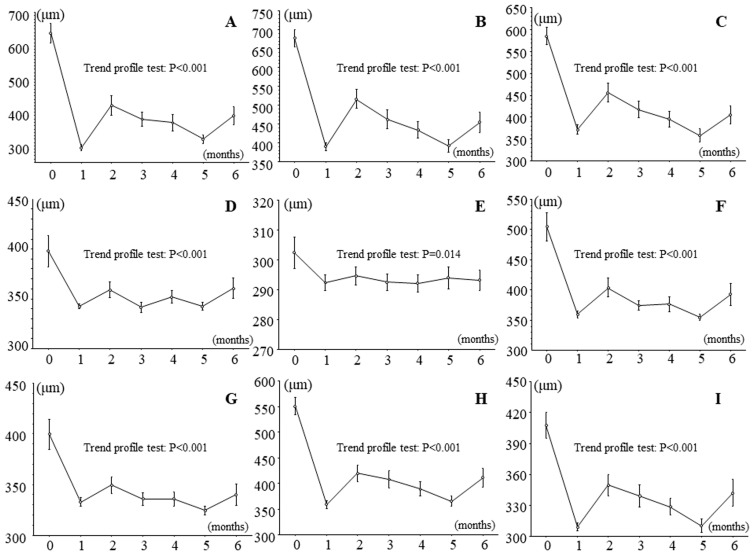
Effects of intravitreal ranibizumab injection on retinal thickness measured by optical coherence tomography in patients with branch retinal vein occlusion and macular edema. Retinal thickness significantly improved in all nine macular subfields in the 6 months after intravitreal ranibizumab injection in patients with branch retinal vein occlusion and macular edema. (**A**) Fovea subfield, (**B**) inner occluded subfield, (**C**) outer occluded subfield, (**D**) inner non-occluded subfield, (**E**) outer non-occluded subfield, (**F**) inner nasal subfield, (**G**) outer nasal subfield, (**H**) inner temporal subfield, (**I**) outer temporal subfield.

**Figure 3:**
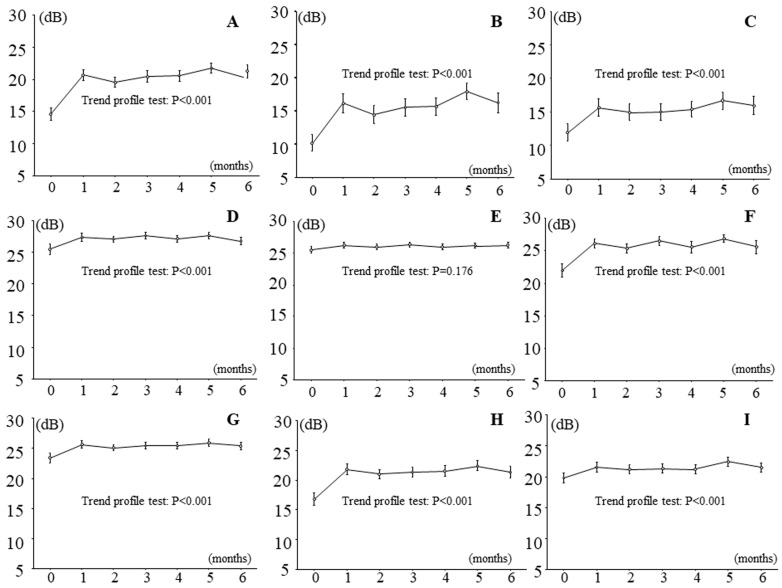
Effects of intravitreal ranibizumab injection on retinal sensitivity measured by MP-3 microperimetry in patients with branch retinal vein occlusion and macular edema. In the 6 months after intravitreal ranibizumab injection, retinal sensitivity significantly improved in 8 retinal regions, i.e., in all regions except (**E**), the outer non-occluded region. (**A**) Fovea region, (**B**) inner occluded region, (**C**) outer occluded region, (**D**) inner non-occluded region, (**E**) outer non-occluded region, (**F**) inner nasal region, (**G**) outer nasal region, (**H**) inner temporal region, (**I**) outer temporal region.

Figure 2 and Figure 3 show the time course of changes in retinal thickness and sensitivity after IRI. Retinal thickness improved significantly in all nine macular subfields during follow-up (Figure 2), and retinal sensitivity significantly improved in eight retinal regions, i.e., in all regions except the outer non-occluded region (Figure 3).

### 3.3. Effects of Intravitreal Ranibizumab Injection on Retinal Thickness and Sensitivity in Patients with Branch Retinal Vein Occlusion and Macular Edema Subdivided into Two Groups According to Improvement in Best Corrected Visual Acuity

**Figure 4:**
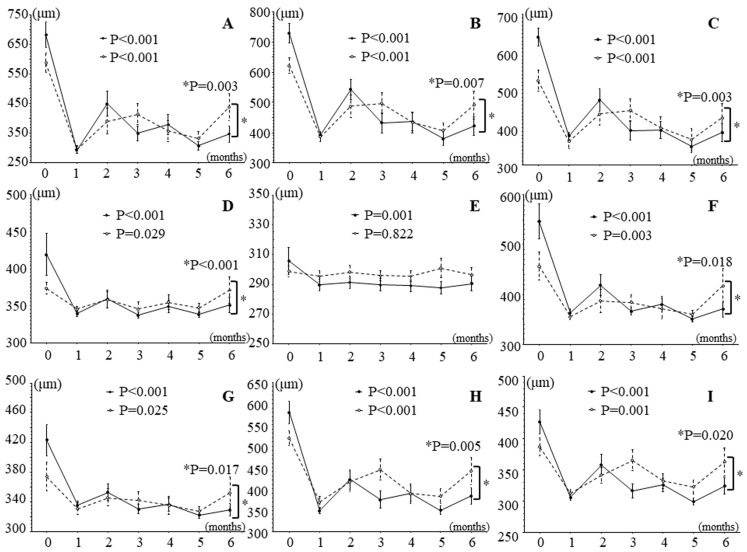
Effects of intravitreal ranibizumab injection on retinal thickness measured by optical coherence tomography in patients with branch retinal vein occlusion and macular edema subdivided into two groups according to improvement in best corrected visual acuity. In patients with good improvement in best corrected visual acuity (BCVA; change in logarithm of minimal angle of resolution best corrected visual acuity > 0.2), retinal thickness showed significant improvements from baseline in all 9 retinal regions, and in patients with poor improvement in BCVA (change in logarithm of minimal angle of resolution best corrected visual acuity < 0.2), retinal thickness showed significant improvements in all regions except (**E**), the outer non-occluded subfield. There were significant differences in the trend profile between the patients with good and poor improvement in BCVA in all regions (*p* values marked with an asterisk in the figure) except in (**E**), the temporal inner subfield (*p* = 0.082). (**A**) Fovea subfield, (**B**) inner occluded subfield, (**C**) outer occluded subfield, (**D**) inner non-occluded subfield, (**E**) outer non-occluded subfield, (**F**) inner nasal subfield, (**G**) outer nasal subfield, (**H**) inner temporal subfield, (**I**) outer temporal subfield. Solid line, patients with good improvement in best corrected visual acuity; dashed line, patients with poor improvement in best corrected visual acuity.

**Figure 5:**
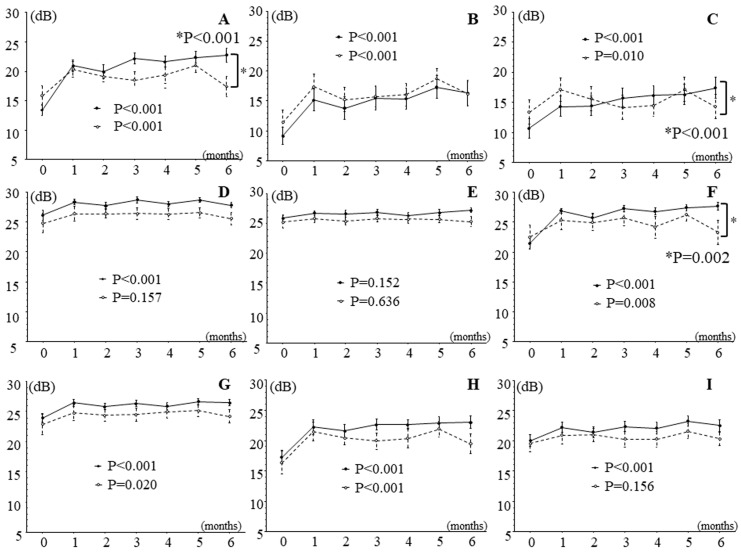
Effects of intravitreal ranibizumab injection on retinal sensitivity measured by MP-3 microperimetry in patients with branch retinal vein occlusion and macular edema subdivided into two groups according to improvement in best corrected visual acuity. In patients with good improvement in best corrected visual acuity (BCVA), retinal sensitivity showed significant improvements in 8 of 9 regions (i.e., in all regions except the outer non-occluded region), and in patients with poor improvement in BCVA, it showed significant improvements in 6 of 9 regions (i.e., not in the inner and outer non-occluded regions or the outer temporal region). In addition, there were significant differences in the trend profile of regions (**A**,**C**,**F**) between patients with good and poor improvement in BCVA (*p* values marked with an asterisk in the figure); however, there were no significant differences in the trend profile of the remaining regions: region (**B**), *p* = 0.792; (**D**), *p* = 0.872; (**E**), *p* = 0.472; (**G**), *p* = 0.732; (**H**), *p* = 0.184; and (**I**), *p* = 0.227. (**A**) Fovea region, (**B**) inner occluded region, (**C**) outer occluded region, (**D**) inner non-occluded region, (**E**) outer non-occluded region, (**F**) inner nasal region, (**G**) outer nasal region, (**H**) inner temporal region, and (**I**) outer temporal region. Solid line, patients with good improvement in best corrected visual acuity; dashed line, patients with poor improvement in best corrected visual acuity.

In patients with good improvement in BCVA (change in logMAR BCVA > 0.2; *n* = 18), IRI significantly improved retinal thickness in all nine macular subfields over time, and in patients with poor improvement in BCVA (change in logMAR BCVA < 0.2; *n* = 16), it significantly improved retinal thickness in eight macular subfields, i.e., in all subfields except the outer non-occluded subfield (Figure 4). In addition, in all subfields except the temporal inner subfield, the trend profile showed significant differences in retinal thickness between patients with good and poor improvement in BCVA (Figure 5).

In patients with good improvement in BCVA (change in logMAR BCVA > 0.2; *n* = 18), IRI significantly improved retinal sensitivity in all regions except the outer non-occluded region during follow-up, and in patients with poor improvement in BCVA (change in logMAR BCVA < 0.2; *n* = 16), it significantly improved retinal sensitivity in six regions, i.e., not in the inner and outer non-occluded regions or the outer temporal region (Figure 5). The trend profile of the foveal, outer occluded, and inner nasal regions also showed significant differences between patients with good and poor improvement in BCVA (Figure 5). 

We found no significant differences between patients with good and poor improvement in BCVA with respect to mean age (59.4 ± 9.1 years vs. 64.8 ± 9.8 years, respectively; *p* = 0.109), sex distribution (10 men and 8 women vs. 6 men and 10 women, respectively; *p* = 0.292), duration of macular edema (28.1 ± 20.8 days vs. 46.0 ± 33.4 days, respectively; *p* = 0.068), prevalence of SRD (8/18 patients vs. 9/16 patients, respectively; *p* = 0.492), or mean number of IRIs (2.6 ± 1.0 vs. 2.9 ± 0.7, respectively; *p* = 0.221).

## 4. Discussion

In this study, we found that visual acuity improved significantly over time after IRI. Retinal thickness improved in all nine macular subfields in the whole group. In patients with good improvement in BCVA, retinal thickness significantly improved also in all nine macular subfields over time, and in patients with poor improvement n BCVA, it significantly improved in eight macular subfields, i.e., in all subfields except the outer non-occluded subfield. Retinal sensitivity improved in eight of the nine retinal regions, i.e., in all except the outer non-occluded region, in the whole group. In patients with good improvement in BCVA, retinal sensitivity improved significantly in all regions except the outer non-occluded region, and in patients with poor improvement in BCVA, it improved significantly in six of the nine regions (i.e., in all regions except the inner and outer non-occluded regions and the outer temporal region). Thus, even in patients with poor improvement in BCVA, retinal sensitivity improved in the majority of regions.

The findings suggest that after performing IRI in patients with BRVO with macular edema, prognosis may be best reflected by a combination of (1) visual acuity (assessed as logMAR BCVA) as a measure of foveal function and (2) macular sensitivity (assessed by MP-3 microperimetry) as a measure of overall retinal sensitivity. Support for this hypothesis is provided by our earlier study, which showed that retinal thickness and volume are associated with visual acuity and retinal sensitivity in BRVO with macular edema [35]. We hypothesize that the improvement in retinal sensitivity could lead to improved QOL, but further investigation is needed to clarify the relationships between retinal sensitivity and QOL.

The study found a significant difference in the trend profile of retinal sensitivity in the foveal region between patients with good and poor improvement in BCVA (Figure 5A). As mentioned before, the measurement of visual acuity primarily reflects foveal function. Therefore, patients with good improvement in BCVA may have had preserved foveal function and improved retinal sensitivity more often than those with poor improvement in BCVA; this topic requires further evaluation in future studies. Furthermore, this study found a significant difference in the trend profile of the outer occluded region between patients with good and poor improvement in BCVA (Figure 5C). In BRVO, the outer occluded region is the area of high retinal hemorrhage and edema, so we hypothesize that the severity of hemorrhage and edema may have affected the retinal sensitivity of the outer occluded region. In fact, the large pre-treatment difference in retinal thickness between the two groups indicates that there may have been a difference in the trend of retinal sensitivity in the outer occluded region (Figure 5C). In addition, this study found a significant difference in the trend profile of the inner nasal region between the patients with good and poor improvement in BCVA (Figure 5F). Previously, we suggested that in BRVO, the sensitivity of the nasal region of the retina (i.e., the papillomacular bundle) tends to decrease less than that of other retinal areas [35]. Support for this suggestion was provided by the finding that numerous retinal ganglion, parasol, and dendritic cells are present in the nasal region of the retina [40]. Therefore, patients with good improvement in BCVA may have had better retinal sensitivity than those with poor improvement because the nasal area function was preserved. Further investigation is needed to clarify these relationships.

Interestingly, we also found that in the 6-month follow-up period after IRI, the retinal thickness significantly improved in all nine regions, including the outer non-occluded region, in all patients and in eight of the nine regions in the subgroup of patients with good improvement in BCVA. We hypothesize that this result may be due to the improvement in edema over the entire macula with anti-VEGF drug treatment by IRI and that it may suggest that the increased vascular permeability in BRVO extends to the entire macula. We also found significant differences in the trend profile of eight regions, i.e., all regions except the outer non-occluded region, between patients with good and poor improvement in BCVA. However, this result may have been significant because there was a large pre-treatment difference in baseline retinal thickness between the two groups (i.e., patients with good improvement in BCVA had a higher baseline retinal thickness).

Our study has several limitations. It was a retrospective study and had only a short follow-up period, so prospective studies with longer follow-up periods are warranted. The study may have lacked power because the sample was relatively small, so the findings need to be confirmed in studies with sufficiently large sample sizes. Only one type of anti-VEGF therapy was studied, and it would be of interest to compare anti-VEGF therapies in a randomized, prospective study. In addition, changes in the external limiting membrane and ellipsoid zone could not be accurately assessed by SD-OCT because of the effects of thick retinal hemorrhages, subretinal hemorrhages, and hard exudates, so improved OCT is needed for use in future research. Additional topics for future study include vision-related QOL in BRVO and potential associations of retinal sensitivity with QOL.

## 5. Conclusions

To our knowledge, this was the first study to assess retinal sensitivity at monthly intervals and to examine differences in the improvement in retinal sensitivity between patients with good and poor visual improvement after any kind of anti-VEGF drug treatment, including IRI. Our results indicate that IRI may significantly improve visual acuity, measured as BCVA, over 6 months. In addition, it may significantly improve the sensitivity in most retinal regions and the thickness of all nine retinal regions; this appears to be the case also in the subset of patients with good improvement in BCVA. In patients with poor improvement in BCVA, IRI also appears to have a positive effect on retinal sensitivity and thickness because, in the present study, sensitivity improved in six regions in this subset of patients, and retinal thickness improved in eight regions. These findings indicate that IRI for macular edema associated with BRVO has beneficial effects on visual acuity and retinal thickness and sensitivity, although the effects may vary by retinal site and may not be as far-reaching in patients with poor visual improvement. Consequently, physicians may have to evaluate visual improvement closely after IRI and may expect to have to repeat IRI more often in patients with less pronounced improvement in visual acuity. 

## Figures and Tables

**Table 1:** Baseline clinical features of patients with macular edema due to branch retinal vein occlusion.

Variables	BRVO (*n* = 34)
Age, years	61.9 ± 9.7 ^‡^
Sex, female/male	18/16
Duration of macular edema, days	37.0 ± 29.0 ^‡^
Hypertension	27 (79.4%)
Systolic blood pressure, mmHg	149 ± 19
Diastolic blood pressure, mmHg	94 ± 14
Hyperlipidemia	16 (47.1%)
Baseline BCVA logMAR, Snellen	0.51 ± 0.36 ^‡^
Baseline CMT, μm	638 ± 166 ^‡^
Presence of SRD	17 (50.0%)

BRVO, branch retinal vein occlusion; BCVA, best-corrected visual acuity; CMT, central macular thickness; log MAR, logarithm of the minimum angle of resolution; SRD, serous retinal detachment; ^‡^ Mean ± standard deviation (SD).

## Data Availability

All data generated or analyzed during this study are included in this article. Further enquiries can be directed to the corresponding author.
